# Novel hypergravity treatment enhances root phenotype and positively influences physio-biochemical parameters in bread wheat (*Triticum aestivum* L.)

**DOI:** 10.1038/s41598-021-94771-8

**Published:** 2021-07-27

**Authors:** Basavalingayya K. Swamy, Ravikumar Hosamani, Malarvizhi Sathasivam, S. S. Chandrashekhar, Uday G. Reddy, Narayan Moger

**Affiliations:** 1 Institute of Agricultural Biotechnology (IABT), University of Agricultural Sciences, Dharwad, Karnataka 580005 India; 2 Department of Seed Science and Technology, University of Agricultural Sciences, Dharwad, Karnataka 580005 India; 3 AICRP on Wheat, University of Agricultural Sciences, Dharwad, Karnataka 580005 India

**Keywords:** Physiology, Plant sciences

## Abstract

Hypergravity—an evolutionarily novel environment has been exploited to comprehend the response of living organisms including plants in the context of extra-terrestrial applications. Recently, researchers have shown that hypergravity induces desired phenotypic variability in seedlings. In the present study, we tested the utility of hypergravity as a novel tool in inducing reliable phenotype/s for potential terrestrial crop improvement applications. To investigate, bread wheat seeds (UAS-375 genotype) were subjected to hypergravity treatment (10×*g* for 12, and 24 h), and evaluated for seedling vigor and plant growth parameters in both laboratory and greenhouse conditions. It was also attempted to elucidate the associated biochemical and hormonal changes at different stages of vegetative growth. Resultant data revealed that hypergravity treatment (10×*g* for 12 h) significantly enhanced root length, root volume, and root biomass in response to hypergravity. The robust seedling growth phenotype may be attributed to increased alpha-amylase and TDH enzyme activities observed in seeds treated with hypergravity. Elevated total chlorophyll content and Rubisco (55 kDa) protein expression across different stages of vegetative growth in response to hypergravity may impart physiological benefits to wheat growth. Further, hypergravity elicited robust endogenous phytohormones dynamics in root signifying altered phenotype/s. Collectively, this study for the first time describes the utility of hypergravity as a novel tool in inducing reliable root phenotype that could be potentially exploited for improving wheat varieties for better water usage management.

## Introduction

Gravity is the only constant fundamental force throughout the evolution of the Earth^1^, hence, all the living organisms are evolutionarily adapted to Earth’s gravity 1×*g*. Any deviation from Earth’s gravity (1×*g*) to either hypergravity or hypogravity causes a fundamental shift in physiology, structure, function, and behavior of organisms including plants^2–6^. Hypergravity is defined as a condition where the force of gravity is more than the Earth’s gravity, and typically expressed as greater than one (> 1×*g*). Hypergravity conditions can be created by customized centrifuges that can precisely regulate acceleration values above 1×*g.* It is reported that a hypergravity treatment can impart phenotypic, physiological, and structural benefits to plants^7,8^. For instance, carrot and rocket salad (*Eruca sativa*) seeds exposed to hypergravity (7×*g*) significantly increased their germination rate, and seedling growth^8,9^. Similarly, chronic hypergravity (at 10×*g* for 8 weeks) was reported to enhance the photosynthesis rate by increasing chloroplast size in *Physcomitrella patens*^10^. Interestingly, seeds exposed to hypergravity conferred salt stress resistance in carrot callus as evident by higher germination and seedling vigor index when compared to salt stress alone^11^.

Hypergravity by virtue of its mechanical loading reported imparting structural strength by manipulating cell wall properties and constituents in peas, cress, and azuki beans^12–14^. In contrast, wheat and rice seeds exposed to acute hypergravity significantly reduced seedling growth, chlorophyll content, photosynthesis rate, transpiration rate, and stomatal conductance^15–17^. In another independent study, wheat seeds subjected to varied hypergravity intensity (500×*g* to 2500×*g* for 10 min) have shown reversible effects including a recovery in germination, and seedling growth parameters in seeds stored for 6 days, and then sown^17^. More recently, maize seeds exposed to 1000×*g*, for 2, 4, and 6 h, exhibited enhanced germination and reduced seedling growth^18^. Thus, these studies indicate hypergravity can significantly induce variability among desired phenotypic traits in both vegetables and field crops. However, many of these studies were narrowly focused and restricted to the seedling stage only and no detailed studies from seedling through the greenhouse conditions from the prism of potential crop improvement have ever been tested.

Here we seek to investigate the utility of hypergravity as a novel tool for inducing desired phenotype/s and physiological traits that can be useful for the potential crop improvement program. To test this hypothesis, we choose wheat (*Triticum aestivum* L., family Poaceae), as it is being hailed as a major global cereal crop, provides staple food for about two billion people (36% of the world’s population) on Earth. In the present study, we carried out a screening study to reach the best intensity and duration that can induce desired phenotypic traits such as enhanced root length and associated phenotypes that may have relevance in drought avoidance or tolerance in wheat. Further, attempts were made to elucidate the biochemical basis including hormonal dynamics of this hypergravity-induced altered phenotype. To the best of our knowledge, this study reports for the first time the utility of hypergravity as a novel tool in inducing desirable phenotypic and physiological traits that have been evaluated for the entire life cycle—from germination to yield under greenhouse conditions for potential crop improvement application.

## Results

### Hypergravity screening studies resulted in optimum intensity and duration that induced significant phenotypic variations at the seedling stage in laboratory conditions

**Figure 1:**
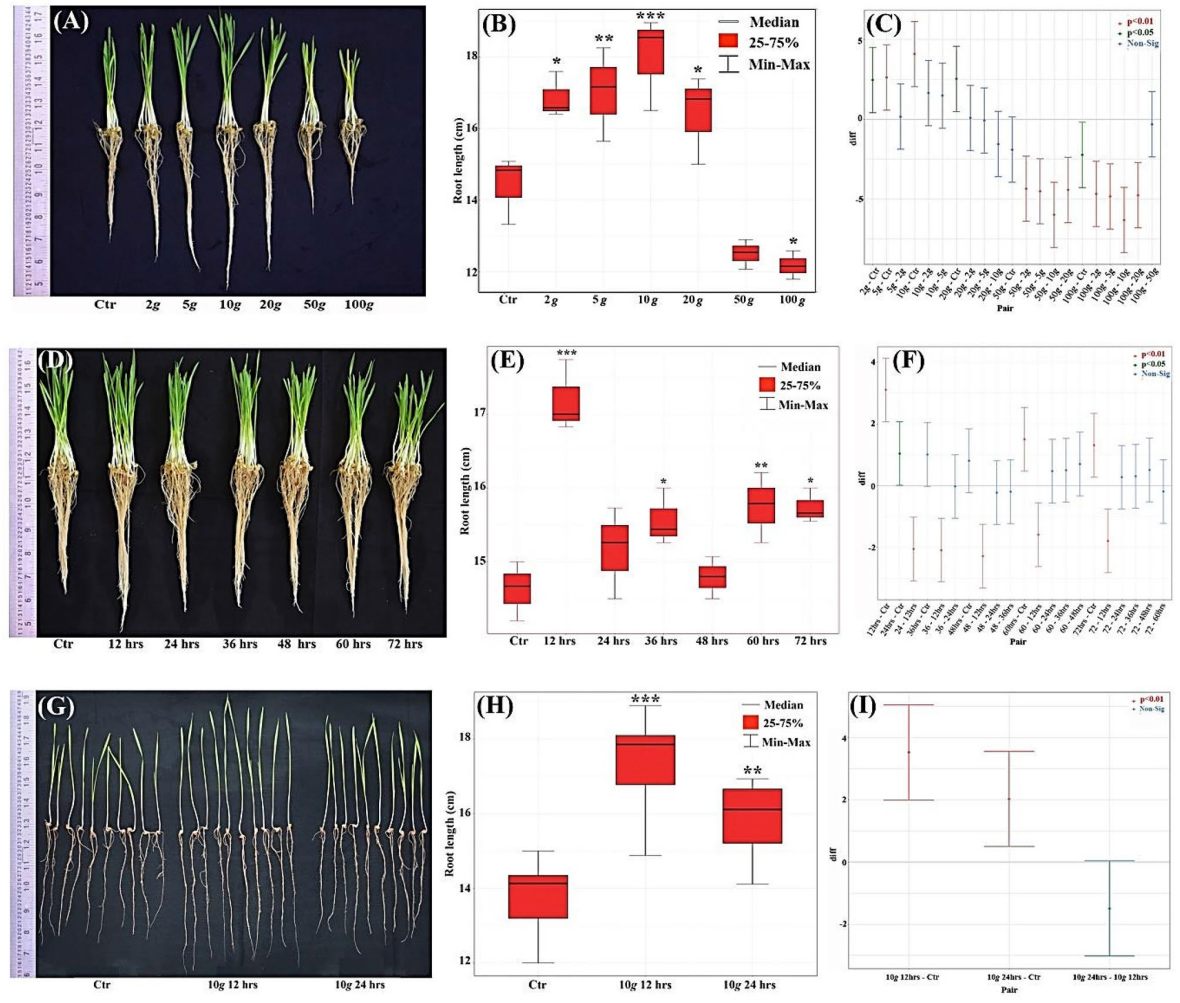
Screening for root and shoot phenotype in wheat using varied hypergravity intensity and duration at the seedling stage in laboratory condition using between-paper-method: (**A**) Qualitative image of root and shoot phenotype response to 2, 5, 10, 20, 50 and 100*×g* for a fixed duration of 12 h; (**B**) Variability (red box plot) among seven treatments (Ctr; 2*×g*; 5*×g*; 10*×g*; 20*×g*, 50*×g*; 100*×g*) of root length phenotype; (**C**) Tukey plot with pairwise comparison with simultaneous 95% confidence level; (**D**) Qualitative images of root and shoot phenotype response to varied hypergravity durations—12, 24, 36, 48, 60 and 72 h for a defined intensity of 10*×g;* (**E**) Variability (red box plot) among seven treatments (Ctr; 12 h; 24 h; 36 h; 48 h; 60 h; 72 h) of root length phenotype; (**F**) Tukey plot with pairwise comparison with simultaneous 95% confidence level; (**G**) Qualitative image of 10*×g,* at 12 and 24 h treatment enhanced root length phenotype; (**H**) Variability (red box plot) among three treatments (Ctr; 10*×g* 12 h; 10*×g* 24 h) of root length phenotype; (**I**) Tukey plot with pairwise comparison with simultaneous 95% confidence level.

For the initial screening study, wheat seeds exposed to 2, 5, 10, 20, 50, and 100×*g* for 12 h duration showed no significant changes in germination rate (79–80%) in any of the above-tested hypergravity intensities. Interestingly, 2, 5, 10 and 20×*g* induced significant increase in root length by 17.00% (p = 0.015), 18.11% (p = 0.009), 28.31% (p = 0.000), and 17.48% (p = 0.012) respectively. In contrast, 50 and 100×*g* inhibited root length by 13.25% (p = 0.074) and 16.03% (p = 0.023) compared to control (Fig. 1A–C). Interestingly, the shoot length phenotype was significantly enhanced by 16.45% (p = 0.002) at 10×*g* only. However, at 50 and 100*×g,* shoot length was significantly decreased by 23.72% (p = 0.000) and 27.09% (p = 0.000) respectively (Table 1).

**Table 1:** Varied hypergravity intensities and durations evaluated to determine the optimum intensity and duration that can induce desired phenotype/s in wheat seedlings in laboratory conditions using between–paper–method (Tab. F (1%) = 4.456 (for intensity and duration study) and 6.013 (for 8th-day).

	Hyper *g* treatment	Mean ± S. Em	% change over control	P ≤ 0.05	Cal. F	CD (5%)	CV (%)
Intensity screening study	**Root length (cm)**						
	Control (1*×g*)	14.41 ± 0.549			33.86	1.782	4.734
	2*×g*	16.86 ± 0.370	17	0.015*			
	5*×g*	17.02 ± 0.756	18.11	0.009*			
	10*×g*	18.49 ± 0.281	28.31	0.000*			
	20*×g*	16.93 ± 0.232	17.48	0.012*			
	50*×g*	12.50 ± 0.237	− 13.25	0.074			
	100*×g*	12.17 ± 0.228	− 16.03	0.023*			
	**Shoot length (cm)**						
	Control (1*×g*)	11.55 ± 0.279			15.68	1.115	4.086
	2*×g*	12.15 ± 0.256	5.10	0.694			
	5*×g*	12.43 ± 0.211	7.53	0.295			
	10*×g*	13.45 ± 0.384	16.45	0.002*			
	20*×g*	11.78 ± 0.249	1.90	0.996			
	50*×g*	08.81 ± 0.168	− 23.72	0.000*			
	100*×g*	08.43 ± 0.253	− 27.09	0.000*			
Duration screening study	**Root length (cm)**						
	Control (1*×g*)	14.25 ± 0.226			19.48	0.900	2.675
	10*×g* 12 h	17.35 ± 0.270	21.72	0.000*			
	24 h	15.29 ± 0.245	7.27	0.049*			
	36 h	14.93 ± 0.187	4.77	0.059			
	48 h	15.06 ± 0.144	5.63	0.181			
	60 h	15.75 ± 0.274	10.52	0.003*			
	72 h	15.56 ± 0.057	9.14	0.010*			
	**Shoot length (cm)**						
	Control (1*×g*)	10.60 ± 0.297			9.963	1.051	4.170
	10*×g* 12 h	11.81 ± 0.496	11.41	0.048*			
	24 h	10.41 ± 0.011	− 1.79	0.998			
	36 h	10.52 ± 0.046	− 0.75	1.000			
	48 h	10.22 ± 0.161	− 3.58	0.928			
	60 h	09.63 ± 0.132	− 9.15	0.156			
	72 h	09.37 ± 0.234	− 11.60	0.045*			
8th day	**Root length (cm)**						
	Control (1*×g*)	13.82 ± 0.304			17.40	1.723	7.150
	10*×g* 12 h	17.34 ± 0.541	25.47	0.000*			
	24 h	15.84 ± 0.392	14.62	0.009*			
	**Shoot length (cm)**						
	Control (1*×g*)	12.53 ± 0.333			6.527	1.707	8.311
	10*×g* 12 h	14.10 ± 0.433	12.53	0.041*			
	24 h	13.42 ± 0.477	07.10	0.312			
	**Seedling vigor index**						
	Control (1*×g*)	2204 ± 96.91			6.574	414.1	7.306
	10*×g* 12 h	2585 ± 104.2	17.29	0.040*			
	24 h	2352 ± 103.2	6.72	0.570			

Having found the highest root and shoot length phenotypic response at 10*×g*, it was decided to test various durations—12, 24, 36, 48, 60, and 72 h with a fixed intensity of 10*×g*. Wheat seeds exposed to varied duration regimens resulted in no significant change in germination rate compared to control groups (79–80%). Among the tested durations, 12, 24, 36 and 60 h induced a significant increase in root length by 21.72% (p = 0.000), 7.27% (p = 0.049), 4.77% (p = 0.059) and 10.52% (p = 0.003) respectively (Fig. 1D–F). Shoot length was increased by 11.49% (p = 0.048) only at 12 h. In contrast, at 72 h time point, there was a significant decrease (by 11.60%; p = 0.045) when compared to control in response to 10*×g* (Table 1). Collectively, a screening study revealed that 10*×g* intensity with 12 and 24 h duration was the best hypergravity regimen as it significantly enhanced root and shoot length in wheat at the seedling stage. Thus, this hypergravity regimen was chosen for all the follow-up studies in this investigation.

### 10×*g* at 12 and 24 h regimen consistently resulted in changes in seedling vigor parameters at the seedling stage (8th-day) in laboratory conditions

To doubly ensure this altered phenotype at 10*×g,* for 12, and 24 h, seven independent experiments with a total of 450 seedlings in each test group were evaluated in germination paper at the seedling stage—8th-day. Consistently as seen in the screening study, here also we found no significant change in germination rate (79–80%) in response to 10*×g* for 12 and 24 h. At 10*×g* for 12 h significantly enhanced root length (25.47%; p = 0.000), shoot length (12.53%; p = 0.041), and seedling vigor index (17.29%; p = 0.040) were observed. However, at 10*×g* for 24 h it was found that significant increase in only root length phenotype (14.62%; p = 0.009), and not shoot length and seedling vigor index (Fig. 1G–I, Table 1).

Additional experiments were conducted to gauze the time-course change in seed germination and root growth rate in laboratory condition. The temporal germination data indicates a significant change in germination rate in response to 10*×g* for 12 h treatment on day 2 only (p = 0.035). Seedling growth rate recorded from first count (4th) to final count (8th day) of seedling stage indicates significant increase in root growth on 7th (p = 0.003) and 8th day (p = 0.006) only. These data points suggest the critical time points to be looked at carefully to elucidate the molecular mechanism/s associated with the altered phenotype (Supplementary Fig. S2, Table S5).

### Greenhouse study: seedling emergence and seedling vigor parameters in response to hypergravity recorded at the seedling stage—8th day

**Figure 2:**
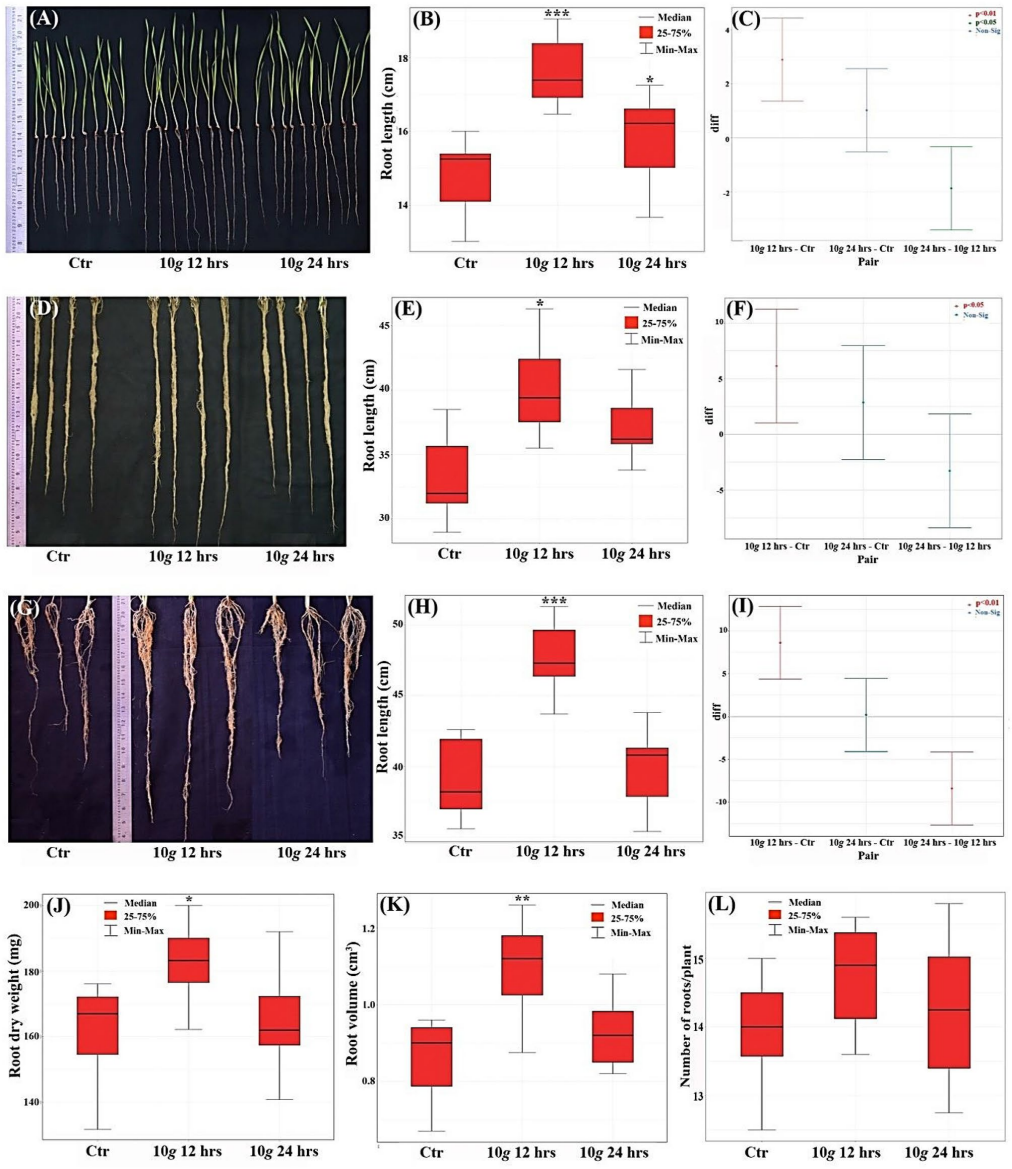
Hypergravity-induced (10*×g,* for 12 and 24 h) enhanced root phenotype recorded at 8th, 20th and 45th-days in a greenhouse condition: (**A**) Qualitative image of root phenotype in response to hypergravity at seedling stage (8th-day); (**B**) Variability (red box plot) among three treatments (Ctr; 10*×g* 12 h; 10*×g* 24 h) of root length phenotype; (**C**) Tukey plot with pairwise comparison with simultaneous 95% confidence level; (**D**) Qualitative image of root phenotype in response to hypergravity at intermediate vegetative stage (20th-day); (**E**) Variability (red box plot) among three treatments (Ctr; 10*×g* 12 h; 10*×g* 24 h) of root length phenotype; (**F**) Tukey plot with pairwise comparison with simultaneous 95% confidence level; (**G**) Qualitative image of root phenotype in response to hypergravity at the beginning of reproductive stage (45th-day); (**H**) Variability (red box plot) among three treatments (Ctr; 10*×g* 12 h; 10*×g* 24 h) of root length phenotype; (**I**) Tukey plot with pairwise comparison with simultaneous 95% confidence level; (**J**) Variability (red box plot) among three treatments (Ctr; 10*×g* 12 h; 10*×g* 24 h) of Root dry weight; (**K**) Root volume in cm^3^; (**L**) Number of roots per plant recorded at 45th-day.

As a natural progression, altered phenotypes induced due to hypergravity treatment were further confirmed in greenhouse conditions in pots. In this study, two independent experiments with four internal replicates with a total of 80 seedlings on the 8th-day were evaluated for seedling emergence, root length, shoot length, seedling dry weight, and seedling vigor index. The 10*×g* at both the time point (12 and 24 h) had no significant impact on seedling emergence (90–95%) in the greenhouse condition. However, root length, shoot length, seedling dry weight and seedling vigor index were significantly enhanced by 19.58% (p = 0.000), 9.15% (p = 0.032), 10.52% (p = 0.049) and 13.73% (p = 0.027) respectively at 10*×g* for 12 h only compared to control. At 10*×g* for 24 h time point no significant change in any of the tested parameters was observed (Fig. 2A–C, Table 2).

**Table 2:** Hypergravity-induced changes in wheat growth parameters recorded on the final count of wheat seedlings (8th-day), at intermediate vegetative growth stage (20th-day), and at the beginning of reproductive phase (45th-day) in greenhouse conditions (Tab. F (5%) = 3.555).

	Hyper *g* treatment	Mean ± S. Em	% change over control	P ≤ 0.05	Cal. F	CD (5%)	CV (%)
8th day	**Root length (cm)**						
	Control (1*×g*)	14.76 ± 0.401			11.83	1.268	7.032
	10*×g* 12 h	17.65 ± 0.371	19.58	0.000*			
	24 h	15.78 ± 0.497	6.91	0.235			
	**Shoot length (cm)**						
	Control (1*×g*)	15.19 ± 0.350			4.059	1.057	5.892
	10*×g* 12 h	16.58 ± 0.302	9.15	0.032*			
	24 h	16.18 ± 0.406	6.52	0.149			
	**Seedling dry weight (mg)**						
	Control (1*×g*)	24.43 ± 0.658			3.667	2.116	7.339
	10*×g* 12 h	27.00 ± 0.887	10.52	0.049*			
	24 h	25.60 ± 0.548	4.79	0.489			
	**Seedling vigor index**						
	Control (1*×g*)	2374 ± 86.44			4.593	240.3	8.324
	10*×g* 12 h	2700 ± 88.01	13.73	0.027*			
	24 h	2638 ± 66.43	11.12	0.080			
20th day	**Root length (cm)**						
	Control (1*×g*)	34.03 ± 1.612			4.681	4.212	10.13
	10*×g* 12 h	40.16 ± 1.462	18.01	0.018*			
	24 h	36.86 ± 1.136	8.31	0.353			
	**Shoot length (cm)**						
	Control (1*×g*)	27.86 ± 0.594			0.116	2.249	11.43
	10*×g* 12 h	28.21 ± 0.924	1.26	0.941			
	24 h	27.71 ± 0.714	− 0.54	0.990			
	**Root volume (cm**^**3**^**)**						
	Control (1*×g*)	0.38 ± 0.039			1.293	0.099	18.06
	10*×g* 12 h	0.41 ± 0.032	6.32	0.093			
	24 h	0.40 ± 0.025	5.26	0.872			
	**Root dry weight (mg)**						
	Control (1*×g*)	24.38 ± 1.205			5.003	4.202	13.74
	10*×g* 12 h	30.61 ± 1.430	25.55	0.015*			
	24 h	26.77 ± 1.581	9.80	0.479			
	**Shoot dry weight (mg)**						
	Control (1*×g*)	49.16 ± 1.872			2.226	6.336	11.45
	10*×g* 12 h	52.59 ± 1.301	5.26	0.505			
	24 h	46.25 ± 2.905	− 5.92	0.604			
45th day	**Root length (cm)**						
	Control (1*×g*)	39.17 ± 2.097			17.25	3.512	13.43
	10*×g* 12 h	47.76 ± 2.982	21.93	0.000*			
	24 h	39.34 ± 1.421	4.07	0.995			
	**Shoot length (cm)**						
	Control (1*×g*)	43.21 ± 2.407			18.94	1.410	12.76
	10*×g* 12 h	48.24 ± 2.202	11.64	0.012*			
	24 h	44.97 ± 2.684	4.10	0.083			
	**Number of roots/plant**						
	Control (1*×g*)	13.84 ± 1.523			0.332	1.898	11.88
	10*×g* 12 h	14.57 ± 1.844	5.31	0.699			
	24 h	14.22 ± 1.486	2.73	0.908			
	**Root volume (cm**^**3**^**)**						
	Control (1*×g*)	0.86 ± 0.045			7.883	0.130	12.11
	10*×g* 12 h	1.10 ± 0.050	27.91	0.004*			
	24 h	0.93 ± 0.037	8.14	0.549			
	**Root dry weight (mg)**						
	Control (1*×g*)	157.3 ± 6.422			4.641	20.69	10.91
	10*×g* 12 h	185.8 ± 7.193	18.09	0.040*			
	24 h	163.4 ± 8.809	3.87	1.000			
	**Shoot dry weight (mg)**						
	Control (1*×g*)	383.9 ± 22.78			1.624	64.90	14.03
	10*×g* 12 h	439.5 ± 23.75	13.18	0.197			
	24 h	412.1 ± 18.65	7.35	0.637			

### Seedling emergence and plant growth parameters in response to hypergravity recorded at the intermediate vegetative stage (20th-day)

To assess the dynamic temporal changes in plant growth phenotypes in response to hypergravity, it was evaluated on the 20th-day—an intermediate vegetative phase of wheat in a pot experiment. In this study, along with seedling emergence, root, and shoot length, additionally, root volume, root dry weight, and shoot dry weight parameters were recorded. Specifically, at 10*×g*, for 12 h time point, root length, and root dry weight were significantly increased by 18.01% (p = 0.018), and 25.55% (p = 0.015) respectively when compared to control. In contrast, no such significant change was observed among these parameters at 24 h time point (Fig. 2D–F). Further, it was observed that shoot length and shoot dry weight were unaffected by the 10*×g* treatment at both 12 and 24 h (Table 2).

### Seedling emergence and plant growth parameters in response to hypergravity recorded at the beginning of the reproductive stage (45th-day)

In the pipe experiment, a total of 35 plants in each test group were divided into seven biological replicates for statistical reliability. In consistent with 20th-day plant growth phenotypes, on 45th-day also, root length and root-associated parameters were significantly enhanced in response to 10*×g*, for 12 h only. In specific, at 10*×g*, for 12 h, root length (Fig. 2G–I), root dry weight (Fig. 2J), and root volume (Fig. 2K), were significantly enhanced by 21.93% (p = 0.000), 18.09% (p = 0.040), and 27.91% (p = 0.004), respectively when compared to control root, however, number of roots per plant increased but it was not statistically significant (Fig. 2L). In contrast, at 24 h, no such significant change in root length and its associated parameters were observed. Further unlike, the 20th-day, at 45th-day, shoot length phenotype was enhanced significantly by 11.64% (p = 0.012) at 10*×g*, for 12 h only. Although seedling emergence, number of tillers, number of roots, and shoot dry weight were recorded, but we found no significant change among these parameters in response to hypergravity at both the time points (Table 2).

Collectively, from the above phenotypic data, root length and root-associated parameters such as root volume and root dry weight were consistently increased at 10*×g*, specifically at 12 h regimen across different vegetative stages of wheat crop (8th, 20th, and 45th-day) in laboratory and greenhouse conditions.

### Wheat seeds exposed to hypergravity resulted in a significant increase in α-amylase, total dehydrogenase (TDH) activities, and altered seed protein profile

**Figure 3:**
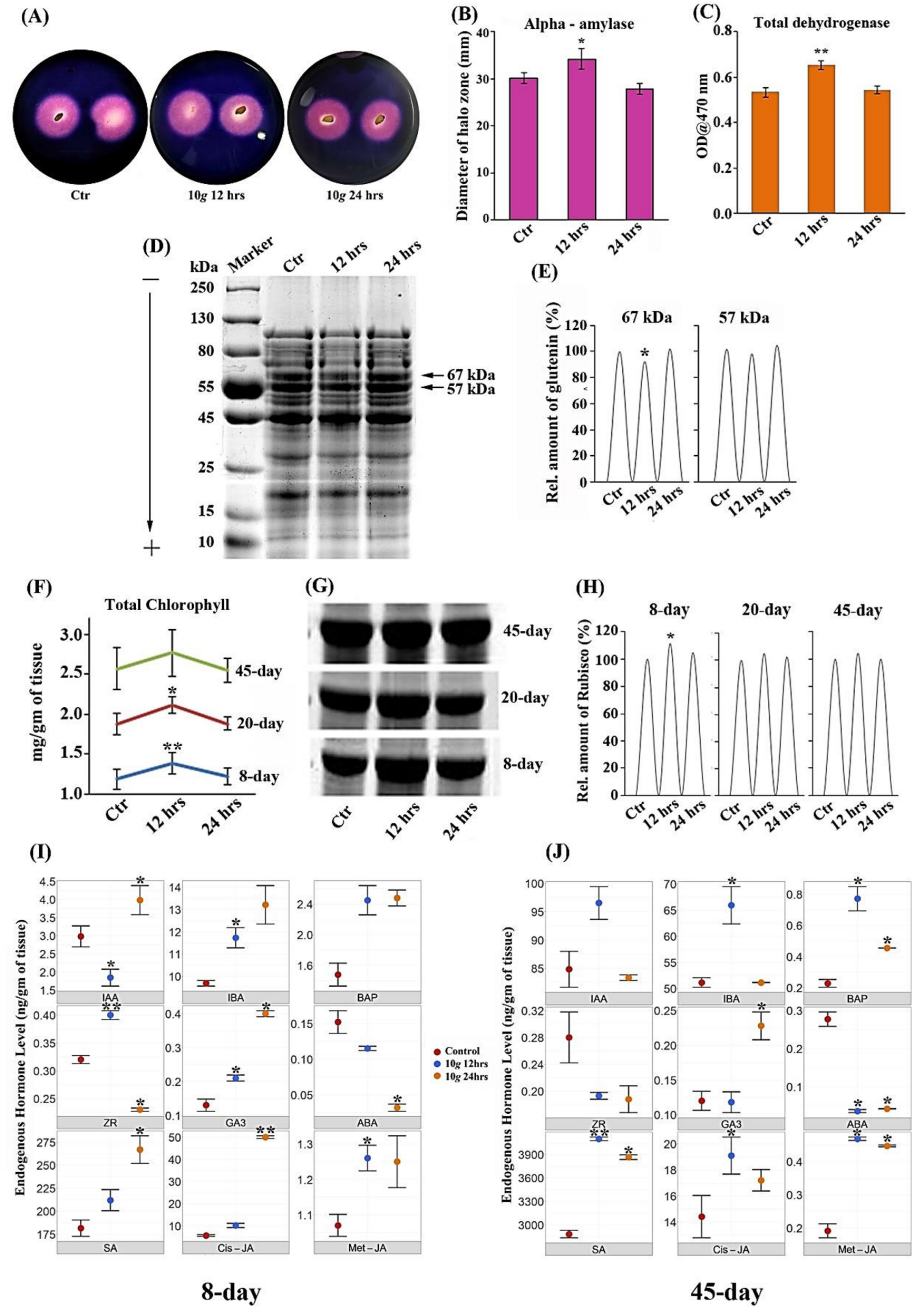
Biochemical parameters assayed post hypergravity treatment: (**A**) representative image of increased mean diameter of halo (clear) zone (in mm) at 10*×g* 12 h indicates significantly enhanced ɑ-Amylase activity in wheat seeds in response to hypergravity; (**B**) Quantitative histogram of amylase activity; (**C**) significant increase in total dehydrogenase (TDH) activity in wheat seeds exposed to hypergravity; (**D**) SDS-PAGE (10%) electrophorogram showing differential banding pattern of wheat seed proteins in response to hypergravity (Lane M: Marker, Lane 1: Control, Lane 2: 10*×g* 12 h, Lane 3: 10×*g* 24 h); (**E**) The qualitatively altered protein band intensity of selected two bands from SDS-PAGE run from wheat seeds exposed to hypergravity was quantified based on relative density; (**F**) A significant increase in total chlorophyll content from leaf tissue isolated from 8th, 20th and 45th-day old wheat plant grown in green house condition post hypergravity treatment; (**G**) Qualitative Rubisco protein band (Cropped and delineated with white space to separate out distinctly from 8th, 20th and 45th-day SDS-PAGE gels run from leaf tissue exposed to hypergravity and control) altered in response to hypergravity at 8th, 20th and 45th-day, full-length gels are presented in Supplementary Fig. S1; (**H**) Quantitative band intensity of Rubisco protein band measured based on relative density; (**I**) Selected phytohormones response to hypergravity measured at 8th-day; (**J**) and 45th-day using UPLC by LC–MS/MS method.

Since seedling vigor was significantly enhanced in response to hypergravity, we decided to investigate the underlying biochemical basis in terms of amylase and TDH activity and protein profile, specifically in seeds, post hypergravity exposure. In response to hypergravity (10*×g* for 12 h) treatment, α-amylase activity was significantly enhanced by 13.57% (p = 0.032) as evident from the increased mean diameter of the halo (clear) zone when compared to untreated seeds. However, at 24 h, no significant change was observed (Fig. 3A,B). Similarly, TDH activity was also significantly enhanced by 22.4% (p = 0.003) at 10*×g*, for 12 h only, with no significant change at 24 h when compared to control seeds (Fig. 3C).

SDS-PAGE (10%) electrophorogram showed a marginal increase in the total number of wheat seed protein bands in response to hypergravity. In specific at 10*×g* for 12 h, and 24 h, nearly the same 25 protein bands (of 10–250 kDa) were found, whereas in control there were 23 protein bands, with a statistically significant difference among them (Fig. 3D). Further, the semi-quantitative band intensity of glutenin protein bands (approximately 67 and 57 kDa) that had shown qualitative changes in band intensity in response to hypergravity was analyzed using the ImageJ program. Interestingly, the 67 kDa (Gluten) protein band intensity was specifically decreased by 7.3% (p = 0.042) at 10*×g* for 12 h only when compared to control. No significant changes were observed in 24 h treatment group and 57 kDa protein bands (Fig. 3D,E). Band intensity was expressed based on relative density after normalization with the control band, 1 (= 100%).

### Hypergravity treatment enhanced total chlorophyll content and Rubisco protein expression quantified at 8th, 20th, and 45th-day of wheat growth in greenhouse conditions

Total chlorophyll content was estimated in all three vegetative growth stages—8th, 20th, and 45th-day in greenhouse conditions. On the 8th and 20th-day, total chlorophyll content was significantly enhanced by 16.65% (p = 0.001) and 12.29% (p = 0.014) respectively in response to 10*×g* for 12 h only, and no significant change was observed in 24 h treatment group. On the 45th day, although a marginal increase in total chlorophyll content (8.23%; p = 0.252) was observed at 12 h, it was not statistically significant. It is interesting to note that as plant growth progressed from the seedling stage (day 8th) to the intermediate vegetative stage (day 20th) to the beginning of the reproductive phase (day 45th) there was a consistent decrease in percent change in total chlorophyll content over control. Further, as anticipated overall total chlorophyll content level increased as plant growth progressed in all the test groups (Fig. 3F).

Interestingly, quantification of Rubisco (55 kDa) band intensity showed a significant increase by 11.5% (p = 0.013) in response to hypergravity treatment (10*×g* for 12 h) at the 8th-day seedling stage only when compared to control. However, Rubisco protein expression on the 20th and 45th-day showed a marginal increase in response to hypergravity, but it was not statistically significant (Fig. 3G,H; gel images were cropped and delineated with white space to separate the 8th, 20th, and 45th-day gels, and full-length gels are presented in Supplementary Fig. S1). It is interesting to note that, enhanced Rubisco (55 kDa) protein at the seedling stage directly correlates with increased chlorophyll content observed in the present study. The band intensity was analyzed using the ImageJ program and expressed based on relative density after normalization with the control band, 1 (= 100%).

### Hypergravity elicits robust phytohormones dynamics in the root

In the present study, 9 major endogenous hormone levels such as auxins, cytokinins, gibberellic acid (GA3), abscisic acid (ABA), salicylic acid (SA), and jasmonic acid (JA) and their derivatives were quantified using the LCMS method at 8th and 45th-day. Among the auxins, indole-3-acetic acid (IAA) was decreased by 38.26% (p = 0.002) in response to 10*×g* 12 h, whereas it was increased by 33.87% (p = 0.012) at 24 h when compared to control. In the case of indole-3-butyric acid (IBA), it was found to increase by 21.2% (p = 0.024) over control only at 10*×g* for 12 h. Among the two tested cytokinins—benzyl aminopurine, and trans-zeatin riboside (ZR), only trans-zeatin riboside was enhanced significantly by 22.89% (p = 0.000) and 29.89% (p = 0.014) at 10*×g *for 12 and 24 h respectively. Similarly, GA3 (gibberellic acid) response to hypergravity revealed interesting dynamism. For instance, GA3 concentration at 12 h was increased by 62.5% (p = 0.013), and at 24 h by 211.7% (p = 0.019) over control. Abscisic acid (ABA) response to hypergravity was significantly negative as evident from decreased concentration by 24.59% (p = 0.021) and 79.02% (p = 0.009) at 10*×g*, for 12, and 24 h respectively over control (Fig. 3I, Supplementary Table S2). Defense hormones such as salicylic acid (SA), cis-jasmonate (cis-JA), methyl-jasmonate (met-JA) showed differential response to both 12 and 24 h of 10*×g *exposure. While salicylic acid and cis-Jasmonate robustly enhanced by 47.06% (p = 0.033) and 791.7% (p = 0.000) respectively at a higher duration (24 h) only, methyl Jasmonate was found to be moderately increased by 17.29% (p = 0.001) and 16.54% (p = 0.049) at 12 and 24 h of 10*×g* treatment respectively over control. Collectively, these stress hormones appear to respond more at 24 h 10*×g* treatment compared to 12 h implying sustained physiological stress imparted by the hypergravity at higher duration with a possible link to negative root phenotype (Fig. 3I, Supplementary Table S2).

Phytohormones quantified at the 45th day induced similar robust dynamics. The auxins such as 3-Indole acetic acid (IAA) and 3-Indole butyric acid (IBA) levels were increased by 13.80% (p = 0.194) and 28.91% (p = 0.031) in response to 10*×g* for 12 h, whereas no significant change was observed at 24 h when compared to control. In the case of cytokinins—benzyl aminopurine (BAP), and trans-zeatin riboside (ZR), the BAP was enhanced significantly by 239.9% (p = 0.035) and 100% (p = 0.013) at 10*×g* for 12 and 24 h respectively. Whereas the ZR was found to be decreased by 31.07% (p = 0.115) and 32.68% (p = 0.259) at 10*×g* for 12 and 24 h respectively when compared to control (Fig. 3J, Supplementary Table S3). Furthermore, the ABA level was significantly reduced by 86.82% (p = 0.004) and 84.66% (p = 0.008) at 10*×g* for 12 and 24 h respectively over the control. The GA3 level was marginally decreased by 1.66% (p = 0.951) at 12 h, while at 24 h, it was significantly enhanced by 90% (p = 0.004) when compared to control. Interestingly, the defense hormones SA, cis-JA, and met-JA showed a similar enhanced response to 12 h and 24 h of 10*×g* exposure as that of 8th-day root hormone dynamics. The salicylic acid was significantly enhanced by 42.26% (p = 0.000) and 34.15% (p = 0.006) at 12 and 24 h respectively. Similarly, the methyl-jasmonate was also robustly increased by 144.6% (p = 0.003) and 133.7% (p = 0.005) at 12 and 24 h of 10*×g* treatment respectively over control. Whereas the cis-jasmonate was found to be significantly increased by 32.57% (p = 0.002) at 12 h exposure only (Fig. 3J, Supplementary Table S3).

### Hypergravity-induced delayed leaf senescence phenotype

**Figure 4:**
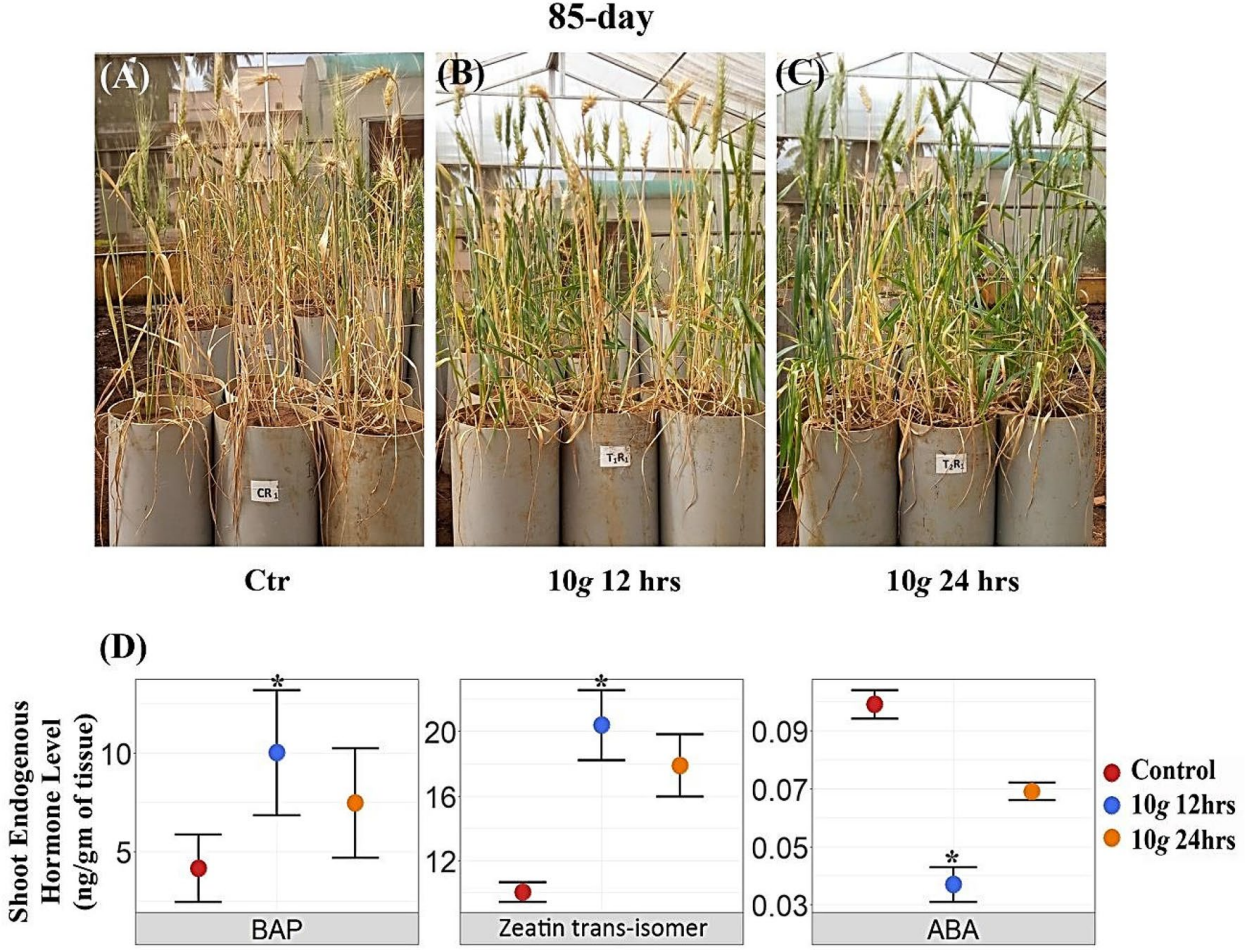
Delayed leaf senescence phenotype induced by hypergravity was observed in greenhouse experiment on 85th-day: (**A**) Control; (**B**) Hypergravity treated—10*×g* for 12 h. **(C)** Hypergravity treated—10*×g* for 24 h; (**D**) Endogenous hormone levels in shoot.

Qualitative observation was taken on the 85th day of standing-crop in the pipe experiment under the greenhouse conditions suggests the delayed leaf senescence phenotype as indicated in the Fig. 4A–C. The qualitative images of plants indicated that hypergravity at 10*×g* exposed for 12 and 24 h retained green leaves longer after anthesis compared to control. The major endogenous hormone level relevant to delayed leaf senescence such as cytokinins (BAP and zeatin trans-isomer) and ABA were quantified in a shoot. Among the cytokinin—BAP and zeatin trans-isomer were significantly enhanced by 140% (p = 0.041) and 103.3% (p = 0.022) respectively at 10*×g* for 12 h exposure. In contrast, the ABA level was significantly reduced by 62.31% (p = 0.000) (Fig. 4D, Supplementary Table S4).

### Hypergravity treatment did not adversely influence yield traits

The selected yield-associated traits such as spike weight (gm), number of spikelets per spike, and 1000 seed weight (gm) were observed and no significant change was found in any of these parameters in response to hypergravity. Interestingly, spike length (cm) significantly increased by 7.68% (p = 0.048) at 10*×g* for 24 h treatment group when compared to control (Table 3).

**Table 3:** Hypergravity treatment did not adversely influence various yield traits (Tab. F (5%) = 3.555).

Plant characters	Treatment	Mean ± S. Em	% change over control	P ≤ 0.05	Cal. F (5%)	CV (%)
Plant height (cm)	Control (1*×g*)	47.39 ± 1.01			0.973	5.427
	10*×g* 12 h	49.31 ± 1.01	4.045	0.380		
	24 h	48.71 ± 0.95	2.775	0.625		
Number of tillers/plant	Control (1*×g*)	04.57 ± 0.20			0.110	11.72
	10*×g* 12 h	04.59 ± 0.18	0.438	0.997		
	24 h	04.70 ± 0.21	2.764	0.901		
Spike length (cm)	Control (1*×g*)	07.31 ± 0.12			3.612	6.12
	10*×g* 12 h	07.29 ± 0.20	− 0.234	0.997		
	24 h	07.89 ± 0.17	7.684	0.048*		
Spike weight (gm)	Control (1*×g*)	01.17 ± 0.07			0.725	12.24
	10*×g* 12 h	01.19 ± 0.04	1.526	0.972		
	24 h	01.26 ± 0.04	7.676	0.504		
Spikelets/spike	Control (1*×g*)	32.80 ± 1.67			0.148	13.41
	10*×g* 12 s	32.96 ± 1.03	0.474	0.998		
	24 h	34.00 ± 2.15	3.643	0.872		
1000 Seeds weight (gm)	Control (1*×g*)	25.94 ± 0.84			0.169	10.76
	10*×g* 12 h	26.81 ± 1.54	3.372	0.834		
	24 h	26.27 ± 0.54	1.292	0.973		

Interestingly spike length at 10*×g* 24 h significantly increased by 7.68% (p = 0.048).

## Discussion

The present study explores the utility of hypergravity for inducing crop improvement traits both at the seedling and vegetative growth of the wheat in laboratory and greenhouse conditions. Initial screening studies identified the optimum hypergravity intensity and duration (10*×g* at 12 and 24 h) regimen that induced enhanced root length, root volume, and root dry weight in wheat. Change in these root traits specifically to 10*×g* for 12 h, might have significantly stimulated columella cells in the root cap and triggered gravisensing signals for increased cell division or growth at the root tip. The gravisensing signal initiates with the physical information derived from the amyloplast sedimentation that can trigger signal transduction to change the direction of auxin transport in the cells. Upon reorientation in root columella cells, PIN-FORMED3 (PIN3), an auxin efflux carrier, re-localises to the bottom side of the cells^19,20^ resulting in the transport of auxin towards the lower side of the reoriented organs. Further, it has been shown that auxin develops an asymmetric distribution in young primary and lateral root tips due to the contributions of PIN3 and PIN7, resulting in a downward growth in response to altered gravity^21^. However, higher hypergravity intensity and duration might have disrupted this signaling pathway leading to inhibition to hypergravitropic response beyond 10*×g* in the present study^19^.

Hypergravity being an added mechanical load; plant cells tend to compact structural components of the cell wall such as cellulose, matrix polysaccharide (hemicellulose and pectin) lignin, and reorientation of cortical microfibrils, thus, resulting in increased lateral growth and reduced longitudinal growth in various plants^13,14,22,23^. For instance, normal and de-capped Azuki bean seedlings under hypergravity conditions exhibited a decrease in the root growth^24^. Similarly, reduced root and shoot growth in wheat seedlings with increased gravity intensity (at 500*×g* to 2500*×g* for 10 min) was reported^25^. In contrast, in the present study, wheat seeds exposed to 10*×g* for 12 h significantly enhanced root length and root volume consistently at seedling and across the different vegetative stages in greenhouse conditions. This differential phenotype can be attributed to the acute and chronic hypergravity protocol followed. Interestingly in the present study, root response to hypergravity was more consistent than shoot. This difference may be attributed to (1) size of vacuoles, unlike root columella cells that have many small vacuoles, shoot endodermal cells contain centrally located large vacuole that occupy > 90% of the total volume ^26^. Hence, shoot endodermal cells may present a significant obstacle^27^ or restricts free fall of gravistimulated amyloplasts. (2) Presence of well-developed thylakoid membrane and photosynthetic pigments in addition to starch granules in shoot endodermal cells; whereas thylakoid membrane system within amyloplasts is completely absent in root columella cells. Lastly, (3) the difference in the arrangement of cytoskeletal structures, unlike root columella cells, shoot endodermal cells contain larger and more compact actin filaments^28^. Hence, compact actin filaments-mediated anchorage of their envelopes at the plasma membrane can prevent large-scale gravity-dependent repositioning of organelles. Collectively, either one or combination of these factors can contribute to the less responsive nature of shoot to hypergravity stimuli compared to root.

Enhanced root length, root volume, root biomass were often correlated with the drought avoidance or tolerance through better water usage management mechanism specifically in rainfed wheat^29–31^. Increased root length was reported to enhance the volume of soil moisture that is available for capture, and root volume substantially increases root surface area and efficiently absorbs water, and imparts drought tolerance^30^. This trait becomes all the more important because wheat being predominantly a non-rainy season crop it needs to ensure better water usage management^32^. At 10*×g* for 12 h, it was also observed that seedling vigor was significantly higher when compared to untreated seeds. Seedling vigor being a critical and yield-defining parameter, enhanced seedling vigor helps in establishing rapid, uniform germination, and robust seedling growth in diverse agro-climatic conditions. This can be partly attributed to the enhanced ɑ-amylase and total dehydrogenase activities in the present study. Increased activities of these two enzymes enable rapid degradation of starch and soluble carbohydrates during seed germination and increased food and gas access to the developing embryos under hypergravity condition. Interestingly, Santos et al*.*^8,9^ and Nunes et al*.*^33^ reported similar robust seedling vigor phenotype upon hypergravity treatment in carrot and rocket salad (7*×g*) and, eucalyptus and corymbia (at 7*×g* for 8 h and 1*×g* for the rest of 16 h for 9 days cycle; uninterrupted hypergravity at 7*×g* and 5*×g* for 8 and 24 h) respectively.

In the present study, total chlorophyll content was significantly enhanced at 10*×g* for 12 h at the initial vegetative stage (8th and 20th-day) and a marginal increase at 45th-day. In line with the findings, *Physcomitrella patens*, a moss-grown in a chronic hypergravity environment (at 10*×g *for 8 weeks) revealed enhanced photosynthesis rate by increasing the chloroplast size^10^. In contrast, wheat seeds exposed to acute hypergravity treatments ranging from 500 to 2500*×g* for 10 min were reported to reduce chlorophyll content coupled with inhibited seedling growth^15,34^. These two contrasting findings may suggest the differential impact of acute and chronic hypergravity protocol. In leaf SDS-PAGE, a specific 55 kDa, Rubisco band (Ribulose-1,5-bisphosphate carboxylase/oxygenase) has shown significant changes in response to hypergravity. This data directly correlates with the increased chlorophyll content implying hypergravity treatment could physiologically benefit the wheat crop.

Seed protein electrophorogram revealed reduced selected band intensity. In general wheat seeds contain 80% gluten proteins with glutenin (67–57 kDa) and gliadin (44–36 kDa) as major fractions^35^. During germination and seedling growth, proteases present in the wheat seed embryo hydrolyze gluten proteins and provides an energy boost for radicle development^36^. Simultaneously, ɑ-amylase and dehydrogenase activities will enhance with reduced gluten content^37^. In consistent with this, in response to hypergravity treatment wheat seeds exhibited increased alpha-amylase and total dehydrogenase enzyme activity coupled with reduced intensity of gluten protein bands. Specifically, the intensity of glutenin fractions (57 and 67 kDa) has reduced when compared to the control group. Thus, boosting the activities of alpha-amylase and TDH leading to enhanced seedling vigor (Fig. 3D).

Phytohormones play a critical role in the growth and morphogenesis of plants. Hence, we investigated the possible role of phytohormones in enhancing root length. Phytohormones profiling at both seedling (8th) and 45th-day-old root samples revealed robust dynamics. Among various phytohormones, the role of natural auxins such as indole-3-acetic acid (IAA) and indole-3-butyric acid (IBA) in regulating root growth are well documented^38–40^. It was reported that IAA strongly decelerates root elongation and the inverse relationship between the endogenous IAA level and root growth rate was confirmed in the elongation zone of maize roots^41^. In contrast, the endogenous level of indole-3-butyric acid (IBA) is shown to have a positive correlation with the regulation of root apical meristem size, root hair elongation, lateral root development, and formation of adventitious roots^42^. In consistent with this, the endogenous level of IAA was significantly reduced, and IBA concentration was increased in response to hypergravity in root samples. ABA is another interesting phytohormone reported to negatively regulate maize root growth similar to IAA^43^. Thus, a positive correlation of IBA and a negative correlation of IAA and ABA were observed with root phenotype.

Zeatin and its derivatives are the important groups of isoprenoid Cytokinins (CKs). Specifically, trans-zeatin riboside (ZR) was reported to possess an antagonistic interaction with ABA as non-hydraulic root-to-shoot signals in drought-stressed and re-watered sunflower plants^44^. In the present study, increased ZR and decreased ABA concentration were observed in response to hypergravity suggesting similar antagonistic interaction between them. A critical role of GA3 in breaking dormancy through the activity of alpha-amylase in rice seeds was reported^45^. Coincidentally, in the present study endogenous GA3 concentration and ɑ-amylase activity were significantly enhanced in hypergravity (10*×g* for 12 h) treated seeds, suggesting a possible link of GA3 for enhanced α-amylase activity leading to robust seedling vigor phenotype. The correlation between root phenotype and endogenous gibberellins (GAs) level can be partly explained by indeterminate effect exerted by gibberellins depending on the concentration as indicated in GA-deficient mutants or GA biosynthesis inhibiting studies^46–50^.

Inhibition of primary root growth by jasmonates is well documented^51–53^. However, it is also reported to influence lateral and adventitious root elongation in *Arabidopsis* and sunflower^51,54^. In consistent with this, jasmonates level was enhanced in response to10×*g* for 12 and 24 h, in which root growth was increased. This may suggest that a certain threshold of jasmonate is critically required for root elongation^55^. Another reason could be increased hypergravity-induced synergy between jasmonate and auxin signaling pathways promoting root regeneration by activating root stem cells as reported by Zhou et al*.*^56^. Similarly, the role of endogenous salicylic acid (SA) in the development of plant roots is relatively unknown. However, the concentration-critical influence of exogenous SA on root growth is reported. For instance, exogenous SA with 250 µM inhibited *Arabidopsis* primary root growth and lateral root development^57^. In contrast, lower SA concentrations induced an increase in root length and biomass in corn at 1.5 µM^58^, in soybean at 100 µM^59^, and in mung bean between 200 and 400 µM. Based on this data, we speculate bioactive concentration window for endogenous SA to induce root growth is critical, and this may have played role in differential response to 10*×g* for 12 and 24 h. Alternatively, robust changes in jasmonate and SA at 10*×g* for 12 and 24 h could be in response to hypergravity-induced physiological stress rather than a direct correlation with root phenotype. Because, jasmonate and SA alone or in combination are known to ameliorate stress conditions^60^, and hypergravity on the other hand is reported to induce such physiological stress in living organism/s including plants^61^.

Another interesting phenotype we observed in our study is delayed leaf senescence on the 85th-day. This phenotype was more prominent at 24 h than 12 h treatment group. It was reported that cytokinins and ABA are known to play a role in delaying leaf senescence in several plant species^62^. Hence, we decided to look at endogenous hormone levels specifically cytokinin and ABA in the shoot. Interestingly, at 12 h treatment, BAP, Zeatin-trans isomer was significantly enhanced and ABA level was decreased in response to hypergravity, thus positively correlating the hormonal dynamics with observed phenotype. This delayed leaf senescence phenotype especially under water stress conditions is significant as it can help in maintaining the remobilization of stored nutrients in source-sink relationships leading to improved crop yield. Collectively, this study, for the first time, indicates that hypergravity can be exploited as a novel tool in inducing desirable phenotype/s and physio-biochemical parameters such as enhanced seedling vigor, root architecture, increased chlorophyll content, and delayed leaf senescence that can be potentially employed for the terrestrial crop improvement program. However, (1) the underlying molecular basis of this altered phenotype/s, (2) how this enhanced root and delayed leaf senescence phenotypes help in imparting drought tolerance, and (3) whether these altered phenotypes are genetically heritable needs to be addressed, and currently, our lab is investigating these questions.

## Methods

### Hypergravity exposure protocol

Hypergravity studies can be carried out using tabletop or floor model centrifuges with cup/tube holders to accommodate seeds. These laboratory centrifuges are in principle similar to the Large Diameter Centrifuge (LDC) facility located at ESTEC (ESA) research center in Noordwijk (The Netherlands) for hypergravity research^63,64^. For this study, we specifically used the floor model high-speed refrigerated centrifuge with a fixed angle rotor A-508C (Model 6000 Lot SER. NO. S40183-G000, Kubota Corporation, Tokyo, Japan). All the hypergravity experiments were carried out at 25 °C temperature maintained inside the centrifuge. Briefly, tubes containing a minimum of 100 good quality wheat seeds (variety UAS-375 were procured from AICRP, wheat scheme unit, University of Agricultural Sciences, Dharwad, Karnataka, India) were exposed to varied hypergravity intensity and durations by spinning at 10*×g* (300 rpm with the radius of rotor 10 cm). The ‘*g*’ force can be calculated using the relative centrifugal force (RCF). The RCF is computed by rotor speed and rotor radius of given centrifuge [*g* = (1.118 × 10 − 5) R × S^2^, where ‘R’ is the radius of the rotor in centimeters (cm), ‘S’ is the speed of the centrifuge in revolutions per minute (rpm) and ‘*g*’ is the relative centrifugal force].

### Phenotype screening study in laboratory conditions

The wheat seeds were exposed to varied hypergravity intensities such as 2, 5, 10, 20, 50, and 100*×g* for 12 h to determine the best hypergravity intensity that can induce the desired seedling phenotype/s. To determine the best treatment duration 12, 24, 36, 48, 60, and 72 h were evaluated. Multiple independent screening experiments were carried out to arrive at the best hypergravity intensity and duration. Based on the literature, to start with we considered desired phenotypes for this screening study were germination and seedling vigor phenotypes—root and shoot length.

### Phenotype screening study in greenhouse conditions

The greenhouse experiments were conducted to confirm the hypergravity-induced changes using polythene bags for the 8th (final count of the seedling stage) and 20th-day (intermediate vegetative stage) studies. And PVC pipes (width 6 inches, 3 feet height) were used for the 45th-day root study (i.e. beginning of the reproductive stage). The polythene bags (50 × 20 cm) with uniformly mixed soil were sown with 8 seeds; two sides of bags were perforated to keep the soil, well-drained. The experiment was laid out in CRD (Completely Randomized Design) with four internal replications of each treatment including control. The same set of experiments was repeated four times for the 8th day’s study, and two times for the 20th-day’s study. Similarly, the plastic pipes with a width of 6 inches with 3 feet in height were used to confirm the hypergravity-induced changes in the wheat root phenotype specifically on the 45th-day. Both polythene bags and pipes were filled with sterilized sand, local black soil (the growth medium), and organic manure in 2:6:2 ratios respectively^65^. The bags/pipes were irrigated regularly with normal water to maintain soil moisture to field capacity. For the pipe experiment total of 12 sets, consisting of 6 pipes in each set was laid out in CRD in seven internal replications of each treatment group. In each pipe, 5 seeds were sown and later thinned out to 2. The phenotypic observations, biochemical changes, and qualitative protein analysis were done on the 8th, 20th, and 45th-days after sowing.

#### Germination rate (%)

The germination test was conducted with 100 seeds in each experiment by following the standard between–paper–method^66^.

#### Shoot and root length (cm)

Seedling vigor parameters such as shoot and root length were determined using standard protocols^67,68^.

#### Seedling vigor index

The seedling vigor index was computed by adopting the method suggested by Abdul Baki and Anderson (1973)^69^ and expressed as an index number. Seedling vigor index = Germination (%) × [Root length (cm) + Shoot length (cm)].

#### Seedling dry weight (mg)

The same set of wheat seedlings used for measuring root and shoot length were kept in butter paper and dried in a hot-air oven maintained at 70 °C temperature for 24 h. Then, the seedlings were allowed to cool in a desiccator for 20 min and the average weight was calculated and expressed in milligram per seedling^70^.

#### A number of roots per plant and root volume (cm^3^)

These two parameters were taken only on the 45th-day. The number of fibrous roots arising from the base of the plant and penetrated the soil was counted as per the standard protocol^70^.

### Physio-biochemical parameters

#### Alpha-amylase and total dehydrogenase (TDH) activities in seeds

The α-amylase activity was analysed as per the method suggested by Simpson and Naylor^71^. Briefly, the pre-soaked (for 8 h) and half-cut seeds were placed on the agar media in such a way that the endospermic part remained in contact with the agar-starch gel. After 48 h of incubation at 30 °C in the dark, potassium iodide solution was uniformly smeared on agar media. After a few minutes, the diameter of the halo (clear) zone formed around the seed was measured in millimetres (mm) and represented as an α-amylase activity.

For assessing total dehydrogenase activity the excised embryos from twenty-five random seeds from each treatment group were steeped into 0.25 percent solution of 2, 3, 5-triphenyl tetrazolium chloride, and incubated in dark for two hours at 40 °C for staining. The stained seeds were washed with water, then soaked in 10 ml of 2 methoxy ethanol (methyl cellosolve), and incubated overnight for extracting the red formazan color. The intensity of red color was measured using a UV–Vis spectrophotometer at 470 nm, and methyl cellosolve was used as a blank. The resultant OD value is represented as total dehydrogenase activity^72^.

#### Total chlorophyll estimation at 8th, 20th, and 45th day

Different photosynthetic pigments such as chlorophyll a, chlorophyll b., and total chlorophylls were estimated using protocol suggested by Bames et al*.*^73^. Briefly, 0.1 g of fresh leaf samples were placed in a test tube containing 10 ml of DMSO and incubated overnight. The chlorophyll extracted into the DMSO solution was collected, and the concentration of chlorophyll a, b, and total chlorophyll were quantified using UV–Vis spectrophotometer at 645 nm and 663 nm using the following formula and represented in mg chl./g of tissue.

$${\text{mg chl}}.{\text{ a}}/{\text{g tissue }} = { 12}.{7 }\left( {\text{A 663}} \right) - {2}.{69 }\left( {\text{A 645}} \right) \times [{\text{V}}/({1}000 \times {\text{W}})],$$

$${\text{mg chl}}.{\text{ b}}/{\text{g tissue }} = { 22}.{9 }\left( {\text{A 645}} \right) - {4}.{68 }\left( {\text{A 663}} \right) \times [{\text{ V}}/({1}000 \times {\text{W}})],$$

$${\text{mg total chl}}./{\text{g tissue }} = { 2}0.{2 }\left( {\text{A 645}} \right) \, + { 8}.0{2 }\left( {\text{A 663}} \right) \times [{\text{V}}/{1}000 \times {\text{W}})].$$

where A = Absorbance at a specific wavelength, V = Final volume of chlorophyll extract in DMSO, W = Fresh weight of tissue extracted.

### Profiling of phytohormones by LCMS: extraction and LC and MS–MS run conditions

The phytohormone profiling study was carried out using the extraction procedure for different plant hormones as described by Pan et al*.*^74^. Briefly, A known quantity (3 gm) of the sample was homogenized using 1-propanol/H_2_O/concentrated HCl (2:1:0.002, v/v/v), sonicated for 30 min at 4 °C, and incubated overnight. The next day, dichloromethane was added to the homogenate, sonicated for 30 min, and then centrifuged at 12,000 rpm for 10 min. Post centrifugation, the bottom aqueous layer was transferred to a conical flask containing sodium sulfate to remove the water traces using a flash evaporator. The dried samples were dissolved in 80% methanol and loaded the sample through C-18 solid-phase extraction (SPE) cartridges to UPLC. The extract is loaded on the preconditioned column and eluted using methanol. The eluted hormones were (5 ml) evaporated to dryness using a flash evaporator with a water bath temperature of 35 °C and dissolved in 500 μl methanol–0.05% formic acid (1:1 v/v). The filtered solution was injected into LCMS. The flow rate was maintained at 0.2 ml/min in the analytical column with 25 °C temperature (2.1 × 50 mm UPLC BEH-C18 column (Waters, USA) with 1.7 μm particles, and protected by a vanguard 2.1 × 5 mm BEH C-18 with 1.7 μm). The eluted hormones were identified and quantified by using an LC–MS/MS (Waters-Acquity, USA) system by comparing the internal standards. For internal standards, all the standard hormones (technical grade) were procured from Sigma-Aldrich and tuned into Mass spectroscopy based on their mass and developed a standard-protocol as follows. Briefly, different concentrations of standard hormones were prepared and injected into LC–MS. From the respective peak area and concentration, the calibration curve was made for each plant hormone and arrived at the standard value (1 peak area = x ng). Later test samples were injected, based on the retention time and mass (particular peak), the hormones were identified and quantified using the standard value and expressed in ng/g of the sample weight. The hormone standards were listed in Supplementary Table S1.

### Qualitative analysis of associated temporal protein changes in response to hypergravity

The qualitative analysis of protein changes associated with hypergravity treatment was carried out using SDS-PAGE. The SDS-PAGE was run with hypergravity treated seeds and leaves obtained from different stages of plant growth on the 8th, 20th, and 45th-day to assess the temporal changes in Rubisco (55 kDa) protein level. The resultant qualitatively altered protein band intensity of selected bands from SDS-PAGE run from wheat seeds and leaf tissue obtained post hypergravity treatment was quantified based on relative density. From leaf tissue SDS-PAGE, specifically, Rubisco protein band cropped from 8th, 20th, and 45th-day SDS-PAGE gels were quantified based on relative density. The experiment was performed with three independent biological replications for statistical reliability. The detailed method of protein extraction^75^, quantification^76^, and SDS-PAGE were carried out as per the standard protocols^77^.

### Yield parameters

To quantify the yield traits at the maturity stage, five plants were taken from each replication and seven independent biological replicates with a total of 35 plants for each group were used. The yield traits such as spike length (cm), spike weight (gm), number of spikelets per spike, and thousand-grain weight (gm) were recorded as per the standard protocols^78^.

### Statistical analysis

All the data generated in this research was at least in triplicates for statistical reliability. The resultant data were subjected to one-way ANOVA with CD at both 1% and 5% level of significance and followed by a post hoc Tukey test to establish multiple pair-wise comparisons using ‘SPSS version 20.0’ and ‘R—version 4.0.2′^79^ software (http://www.R-project.org/). The data collected from the biochemical and qualitative protein analysis experiments were subjected to the independent variable ‘T’ test.

### Ethical approval

Wheat seeds (UAS-375 genotype) collected from AICRP on Wheat, University of Agricultural Sciences, Dharwad, by obtaining due permission from the authority.

All research experiments on wheat carried out in both laboratory and greenhouse conditions in the present investigation strictly followed the guidelines of the University of Agricultural Sciences, Dharwad, Karnataka, India.

## Supplementary Information

Supplementary Information.
