# Supplementary material for: Novel hypergravity treatment enhances root phenotype and positively influences physio-biochemical parameters in bread wheat (Triticum aestivum L.)
Source: Sci Rep. 2021 Jul 27;11:15303. doi: 10.1038/s41598-021-94771-8 (PMC8316474; doi:10.1038/s41598-021-94771-8)
Supplement: Supplementary file 1 — Supplementary Information. [file 41598_2021_94771_MOESM1_ESM.doc]

**REVISED VERSION**

**Novel hypergravity treatment enhances root phenotype and positively influences physio-biochemical parameters in bread wheat (*Triticum aestivum* L.)**

Basavalingayya K Swamy ^1 #^, Ravikumar Hosamani ^1 #^✉, Malarvizhi Sathasivam ^1^, Chandrashekhar S.S ^2^ , Uday Reddy G^3^ and Narayan Moger ^1^

^1^ Institute of Agricultural Biotechnology (IABT), ^2^ Department of Seed Science and Technology, ^3^AICRP on Wheat, University of Agricultural Sciences, Dharwad Karnataka– 580005, India

**^#^** shared first author


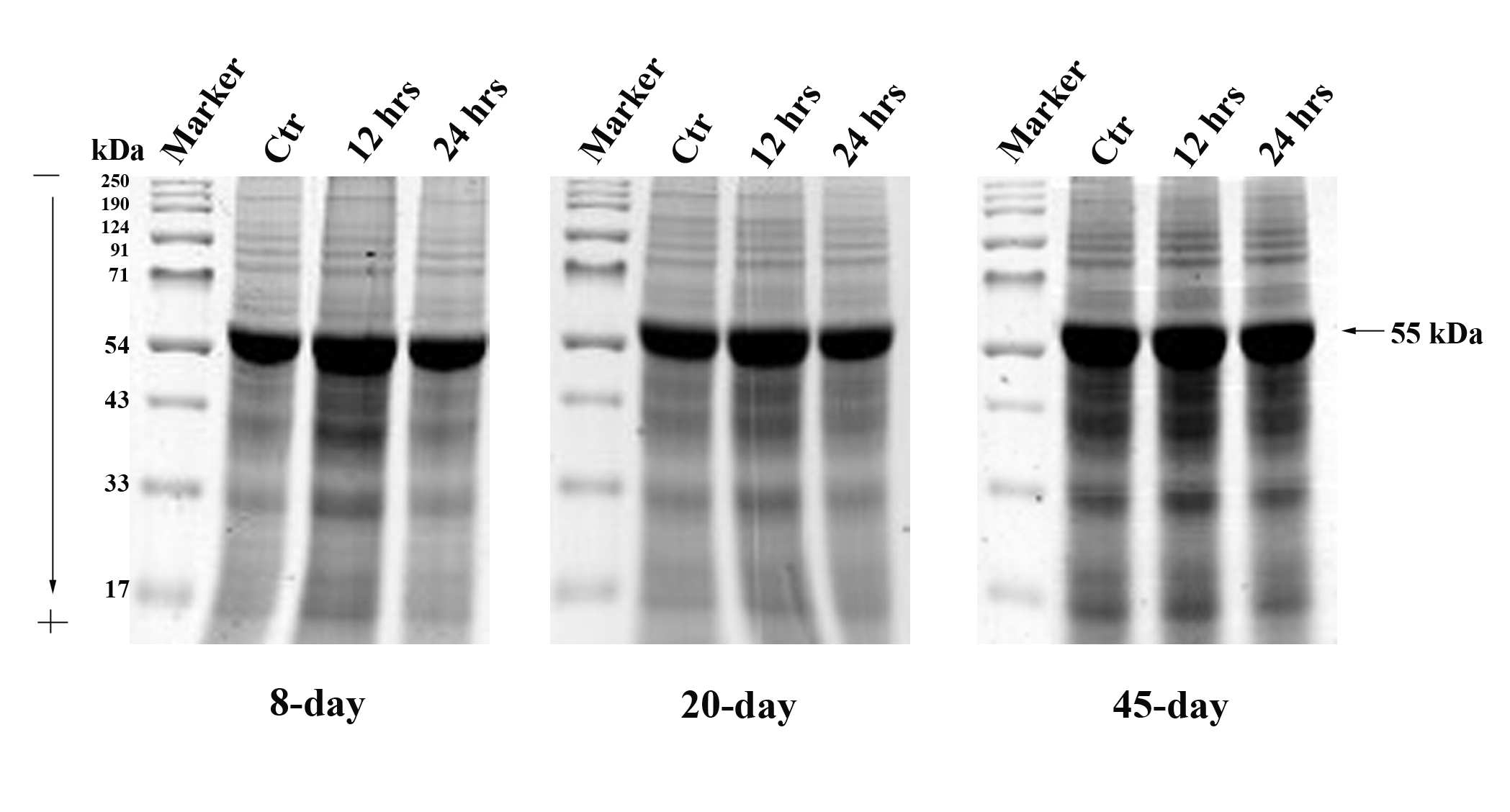


**Supplementary Figure: S1.** SDS-PAGE (10%) electrophorogram showing differential banding pattern of wheat shoot proteins in response to hypergravity isolated from the 8^th^, 20^th^ and 45^th^-day wheat grown in a greenhouse. The qualitatively altered Rubisco (55 kDa) protein is arrow marked. Cropped gel indicating the Rubisco (55 kDa) protein band is presented in **Figure 3, Panel G**

**
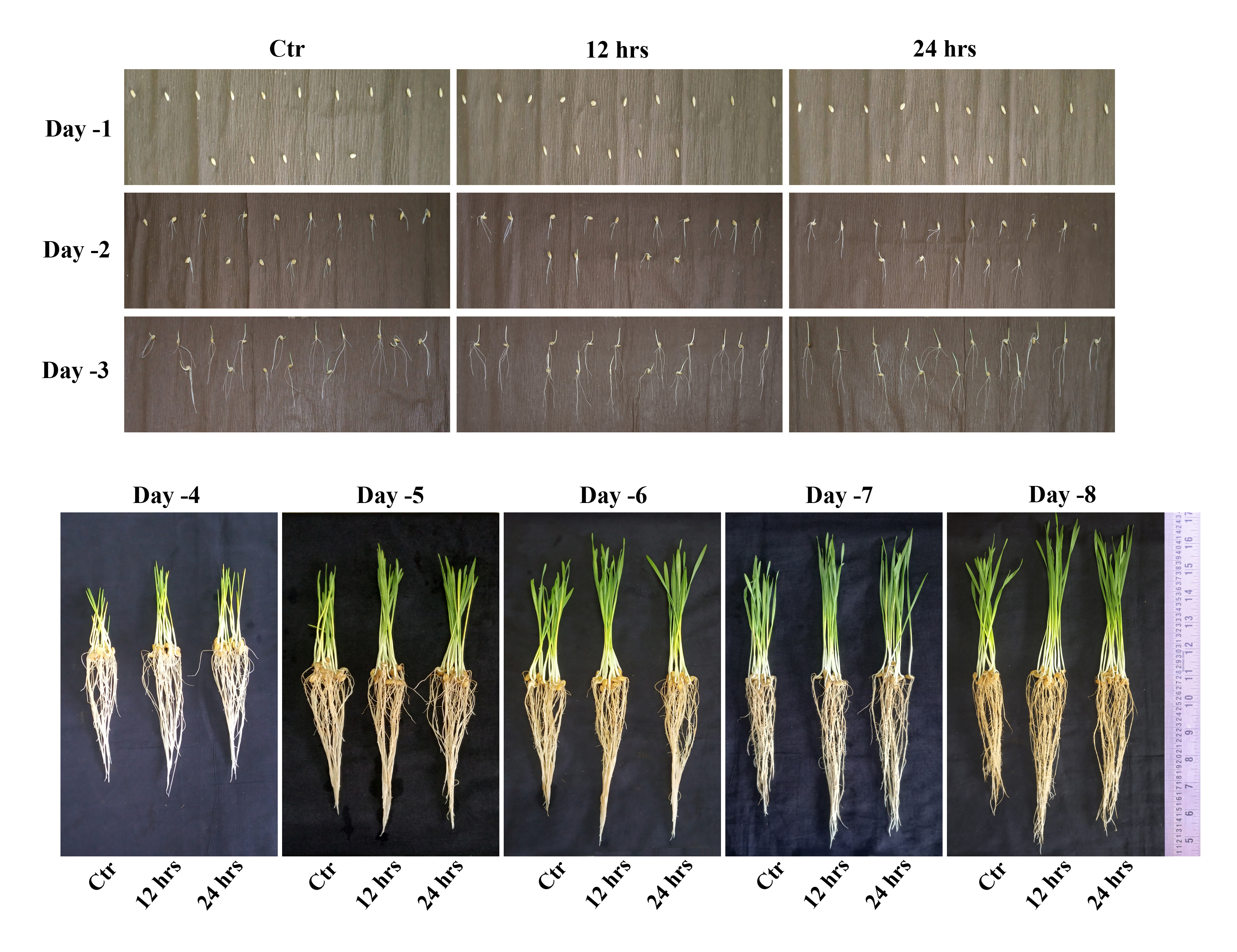
**

**Supplementary Figure: S2.** Time-course observation (**Day 1 to 8**) of hypergravity-induced changes in seed germination and seedling growth rate parameters after seed imbibition in laboratory conditions

| **Sl. No.** | **Compounds** | **Formula/ Mass** | **Parent** **ion (m/z)** | **Cone** **voltage** **(V)** | **Collision energy** **(eV)** | **Ion** **mode** |
| --- | --- | --- | --- | --- | --- | --- |
| 1 | Trans Zeatin riboside (ZR) | 220.2 | 221.4 | 29 | 25 | ES+ |
| 2 | Zeatin trans isomer | 219.1 | 220.13 | 18 | 14 | ES+ |
| 3 | Cis-Jasmonate | 164.0 | 165.03 | 10 | 8 | ES+ |
| 4 | Methyl-Jasmonate | 224.0 | 225.03 | 8 | 12 | ES+ |
| 5 | Salicylic acid (SA) | 138.0 | 137.03 | -14 | -8 | ES- |
| 6 | Abscisic Acid (ABA) | 264.0 | 263.22 | -20 | -8 | ES- |
| 7 | Gibberellic acid (GA3) | 345.9 | 345.00 | -22 | -20 | ES- |
| 8 | Indole Acetic Acid (IAA) | 175.0 | 173.97 | -14 | -10 | ES- |
| 9 | Indole Butyric Acid (IBA) | 203.0 | 201.90 | -22 | -10 | ES- |
| 10 | Benzyl amino purine (BAP) | 121.0 | 122.3 | -18 | -14 | ES- |

**Supplementary Table: S1.** List of hormone standards (technical grade) procured from Sigma-Aldrich were used as an internal standard.

| **3-Indole acetic acid (IAA)** | | | | | |
| --- | --- | --- | --- | --- | --- |
| **Hyper *g* treatment** | **ng/gm of tissue (Mean ± S. Em)** | **% change over control** | **SD** | **t-stat.** | **P(T<=t)**  **Two tail** |
| Control (1*g*) | 2.98 ± 0.290 |  |  |  |  |
| 10*g* 12 hrs | 1.84 ± 0.234 | -38.26 | 0.10 | 20.22 | 0.002* |
| 24 hrs | 3.98 ± 0.403 | 33.87 | 0.20 | 8.95 | 0.012* |
| **3-Indole butyric acid (IBA)** | | | | | |
| Control (1*g*) | 09.68 ± 0.128 |  |  |  |  |
| 10*g* 12 hrs | 11.73 ± 0.454 | 21.20 | 0.57 | 6.29 | 0.024* |
| 24 hrs | 13.21 ± 0.864 | 36.51 | 1.72 | 4.55 | 0.071 |
| **Benzyl aminopurine (BAP)** | | | | | |
| Control (1*g*) | 1.48 ± 0.157 |  |  |  |  |
| 10*g* 12 hrs | 2.45 ± 0.191 | 65.30 | 0.60 | 2.77 | 0.109 |
| 24 hrs | 2.48 ± 0.104 | 67.32 | 0.45 | 3.80 | 0.063 |
| **Trans-zeatin Riboside** | | | | | |
| Control (1*g*) | 0.32 ± 0.008 |  |  |  |  |
| 10*g* 12 hrs | 0.40 ± 0.008 | 22.89 | 0.001 | 258 | 0.00* |
| 24 hrs | 0.23 ± 0.004 | 29.89 | 0.02 | 8.22 | 0.014* |
| **Gibberellic acid 3 (GA3)** | | | | | |
| Control (1*g*) | 0.13 ± 0.018 |  |  |  |  |
| 10*g* 12 hrs | 0.21 ± 0.092 | 62.5 | 0.016 | 8.66 | 0.013* |
| 24 hrs | 0.40 ± 0.005 | 211.7 | 0.048 | 9.77 | 0.019* |
| **Abscisic acid (ABA)** | | | | | |
| Control (1*g*) | 0.152 ± 0.016 |  |  |  |  |
| 10*g* 12 hrs | 0.115 ± 0.003 | -24.59 | 0.022 | 2.88 | 0.021* |
| 24 hrs | 0.032 ± 0.005 | -79.02 | 0.019 | 10.7 | 0.009* |
| **Salicylic acid (SA)** | | | | | |
| Control (1*g*) | 181.5 ± 0.880 |  |  |  |  |
| 10*g* 12 hrs | 212.0 ± 11.44 | 16.81 | 21.35 | 2.47 | 0.132 |
| 24 hrs | 266.9 ± 14.97 | 47.06 | 27.45 | 5.38 | 0.033* |
| **Cis-Jasmonate** | | | | | |
| Control (1*g*) | 05.60 ± 0.395 |  |  |  |  |
| 10*g* 12 hrs | 10.13 ± 1.619 | 80.62 | 2.12 | 3.69 | 0.066 |
| 24 hrs | 50.01 ± 0.603 | 791.7 | 0.36 | 213.3 | 0.00* |
| **Methyl Jasmonate** | | | | | |
| Control (1*g*) | 1.07 ± 0.032 |  |  |  |  |
| 10*g* 12 hrs | 1.26 ± 0.037 | 17.29 | 0.008 | 37.79 | 0.001* |
| 24 hrs | 1.25 ± 0.074 | 16.54 | 0.072 | 4.24 | 0.049* |

**Supplementary Table: S2.** Hypergravity elicits robust phytohormones dynamics in the root, profiled on the final count of wheat seedlings (8^th^-day) grown in greenhouse conditions **(Tab.t = 4.303)**

| **3-Indole acetic acid (IAA)** | | | | | |
| --- | --- | --- | --- | --- | --- |
| **Hyper *g* treatment** | **ng/gm of tissue (Mean ± S. Em)** | **% change over control** | **SD** | **t-stat.** | **P(T<=t)**  **Two tail** |
| Control (1*g*) | 84.82 ± 3.814 |  |  |  |  |
| 10*g* 12 hrs | 96.53 ± 2.90 | 13.8 | 10.54 | 1.92 | 0.194 |
| 24 hrs | 83.33 ± 0.493 | -1.75 | 06.37 | 0.41 | 0.725 |
| **3-Indole butyric acid (IBA)** | | | | | |
| Control (1*g*) | 51.13 ± 0.915 |  |  |  |  |
| 10*g* 12 hrs | 65.92 ± 3.57 | 28.91 | 4.61 | 5.54 | 0.031* |
| 24 hrs | 51.11 ± 0.025 | -0.03 | 1.54 | 0.02 | 0.984 |
| **Benzyl aminopurine (BAP)** | | | | | |
| Control (1*g*) | 0.227 ± 0.025 |  |  |  |  |
| 10*g* 12 hrs | 0.771 ± 0.078 | 239.9 | 0.18 | 5.19 | 0.035* |
| 24 hrs | 0.454 ± 0.000 | 100 | 0.04 | 8.73 | 0.013* |
| **Trans-zeatin Riboside** | | | | | |
| Control (1*g*) | 0.28 ± 0.038 |  |  |  |  |
| 10*g* 12 hrs | 0.193 ± 0.005 | -31.07 | 0.05 | 2.69 | 0.115 |
| 24 hrs | 0.188 ± 0.020 | -32.68 | 0.10 | 1.56 | 0.259 |
| **Gibberellic acid 3 (GA-3)** | | | | | |
| Control (1*g*) | 0.12 ± 0.013 |  |  |  |  |
| 10*g* 12 hrs | 0.118 ± 0.015 | -1.66 | 0.05 | 0.06 | 0.951 |
| 24 hrs | 0.228 ± 0.020 | 90 | 0.01 | 15.5 | 0.004* |
| **Abscisic acid (ABA)** | | | | | |
| Control (1*g*) | 0.277 ± 0.019 |  |  |  |  |
| 10*g* 12 hrs | 0.036 ± 0.004 | -86.82 | 0.025 | 16.33 | 0.004* |
| 24 hrs | 0.042 ± 0.001 | -84.66 | 0.036 | 11.12 | 0.008* |
| **Salicylic acid (SA)** | | | | | |
| Control (1*g*) | 2880.4 ± 48.87 |  |  |  |  |
| 10*g* 12 hrs | 40.97.6 ± 22.45 | 42.26 | 45.75 | 46.7 | 0.00* |
| 24 hrs | 3864.2 ± 30.38 | 34.15 | 137.2 | 12.41 | 0.006* |
| **Cis-Jasmonate** | | | | | |
| Control (1*g*) | 14.4 ± 1.628 |  |  |  |  |
| 10*g* 12 hrs | 19.09 ± 1.420 | 32.57 | 0.36 | 22.56 | 0.002* |
| 24 hrs | 17.2 ± 0.822 | 19.48 | 1.39 | 3.48 | 0.073 |
| **Methyl Jasmonate** | | | | | |
| Control (1*g*) | 0.191 ± 0.021 |  |  |  |  |
| 10*g* 12 hrs | 0.468 ± 0.005 | 144.6 | 0.028 | 17.13 | 0.003* |
| 24 hrs | 0.447 ± 0.003 | 133.7 | 0.032 | 13.85 | 0.005* |

**Supplementary Table: S3.** Hypergravity elicits robust phytohormones dynamics in the root, profiled on the 45^th^-day grown in greenhouse conditions **(Tab.t = 4.303)**

| **Benzyl aminopurine (BAP)** | | | | | |
| --- | --- | --- | --- | --- | --- |
| **Hyper *g* treatment** | **ng/gm of tissue (Mean ± S. Em)** | **% change over control** | **SD** | **t-stat.** | **P(T<=t)**  **Two tail** |
| Control (1*g*) | 4.17 ± 1.711 |  |  |  |  |
| 10*g* 12 hrs | 10.02 ± 4.179 | 140 | 4.27 | 2.37 | 0.041* |
| 24 hrs | 7.47 ± 2.777 | 78 | 1.84 | 3.09 | 0.09 |
| **Zeatin trans isomer** | | | | | |
| Control (1*g*) | 10.03 ± 0.602 |  |  |  |  |
| 10*g* 12 hrs | 20.39 ± 2.176 | 103.3 | 2.72 | 6.58 | 0.022* |
| 24 hrs | 17.89 ± 1.935 | 78.33 | 4.39 | 3.09 | 0.09 |
| **Abscisic acid (ABA)** | | | | | |
| Control (1*g*) | 0.099 ± 0.005 |  |  |  |  |
| 10*g* 12 hrs | 0.037 ± 0.006 | -62.31 | 0.002 | 53.69 | 0.00* |
| 24 hrs | 0.069 ± 0.003 | -30.15 | 0.015 | 3.46 | 0.074 |

**Supplementary Table: S4.** Hypergravity-induced phytohormones level changes in shoot correlating with the delayed leaf senescence phenotype **(Tab.t = 4.303)**

| **Germination per cent (Day 1 to 4)** | | | | | | |
| --- | --- | --- | --- | --- | --- | --- |
| **Germination rate (%)** | **Day-1** | | | | | |
|  | **Hyper *g* treatment** | **Mean ± S. Em** | **% change over control** | | **t-stat.** | **P(T<=t)**  **Two tail** |
|  | Control (1*g*) | 0 | - | | - | - |
|  | 10*g* 12 hrs | 0 |  |  |  |  |
|  | 24 hrs | 0 |  |  |  |  |
|  | **Day-2** | | | | | |
|  | Control (1*g*) | 68.88 ± 5.879 |  | |  |  |
|  | 10*g* 12 hrs | 88.88 ± 5.879 | 20.00 | | 5.196 | 0.035* |
|  | 24 hrs | 82.22 ± 8.012 | 13.34 | | 1.732 | 0.225 |
|  | **Day-3** | | | | | |
|  | Control (1*g*) | 100 | - | | - | - |
|  | 10*g* 12 hrs | 100 |  |  |  |  |
|  | 24 hrs | 100 |  |  |  |  |
|  | **Day-4** | | | | | |
|  | Control (1*g*) | 100 | - | | - | - |
|  | 10*g* 12 hrs | 100 |  |  |  |  |
|  | 24 hrs | 100 |  |  |  |  |
| **Root growth rate (Day 4 to 8)** | | | | | | |
| **Root length (cm)** | **Day-4** | | | | | |
|  | Control (1*g*) | 10.00 ± 0.152 |  | |  |  |
|  | 10*g* 12 hrs | 10.49 ± 0.531 | 4.90 | | 2.944 | 0.099 |
|  | 24 hrs | 10.12 ± 0.105 | 1.20 | | 0.901 | 0.463 |
|  | **Day-5** | | | | | |
|  | Control (1*g*) | 11.13 ± 0.352 |  |  | |  |
|  | 10*g* 12 hrs | 11.95 ± 0.394 | 7.36 | | 1.668 | 0.237 |
|  | 24 hrs | 11.48 ± 0.303 | 3.14 | | 4.041 | 0.056 |
|  | **Day-6** | | | | | |
|  | Control (1*g*) | 12.36 ± 0.375 |  | |  |  |
|  | 10*g* 12 hrs | 13.95 ± 0.394 | 12.83 | | 2.241 | 0.154 |
|  | 24 hrs | 13.98 ± 0.245 | 8.22 | | 2.911 | 0.101 |
|  | **Day-7** | | | | | |
|  | Control (1*g*) | 13.56 ± 0.375 |  | |  |  |
|  | 10*g* 12 hrs | 15.48 ± 0.323 | 14.15 | | 19.46 | 0.003* |
|  | 24 hrs | 15.05 ± 0.076 | 10.93 | | 4.572 | 0.045* |
|  | **Day-8** | | | | | |
|  | Control (1*g*) | 14.2 ± 0.346 |  | |  |  |
|  | 10*g* 12 hrs | 16.6 ± 0.161 | 17.04 | | 13.099 | 0.006* |
|  | 24 hrs | 15.9 ± 0.444 | 12.18 | | 7.626 | 0.023* |

**Supplementary Table: S5.** A significant change in germination and root growth in response to 10*g* for 12 hrs treatment was observed at day 2 (germination rate) and 7^th^ and 8^th^ day (root length) suggesting the critical time point. Germinated seeds were noted daily until the first count of the wheat seedling test (4^th^-day). Seeds were considered germinated when the radicle showed at least 2 mm in length (supplementary figure: S2) and the germination per cent and root growth were computed according to ISTA. **(Tab.t = 4.303).**
